# Retraction: Butyrate Attenuates Lipopolysaccharide-Induced Inflammation in Intestinal Cells and Crohn's Mucosa through Modulation of Antioxidant Defense Machinery

**DOI:** 10.1371/journal.pone.0224849

**Published:** 2019-10-30

**Authors:** The PLOS ONE Editors

After publication of [1] the following concerns were raised:

- Fig 1B and 2C β-actin panels appear similar;
- Fig 3A p-p65 panel contains a vertical discontinuity between lanes 2 and 3.
- In the Fig 3A αβ tubulin panel, the background appears different in lanes 1, 3 than in lanes 2, 4. Lanes 1 and 3 appear similar when inverted horizontally, and lanes 2, 4 appear similar.
- In the Fig 3A Lamin B panel, lanes 1, 3 appear similar and lanes 2, 4 appear similar, and there appear to be vertical discontinuities between lanes.

The authors were unable to provide underlying primary data to resolve the concerns.

In light of the concerns affecting multiple figure panels that question the integrity of these data, the *PLOS ONE* Editors retract this article.

IR and CC disagree with the retraction and stand by the results of the study. AL, PDC, and ET did not respond or could not be reached.
